# Aggressive Thromboprophylaxis Improves Clinical Process and Decreases the Need of Intensive Care Unit in Covid-19

**DOI:** 10.12669/pjms.37.3.3687

**Published:** 2021

**Authors:** Murat Ugur, Esra Adiyeke, Eymen Recep, Nurten Bakan, Nurettin Yiyit

**Affiliations:** 1 Murat Ugur, Department of Cardiovascular Surgery, Health Sciences University, Sancaktepe Sehit Professor Doctor Ilhan Varank Education and Research Hospital, Istanbul, Turkey; 2 Esra Adiyeke, Department of Anesthesiology and Reanimation, Health Sciences University, Sancaktepe Sehit Professor Doctor Ilhan Varank Education and Research Hospital, Istanbul, Turkey; 3 Eymen Recep, Department of Cardiovascular Surgery, Health Sciences University, Sancaktepe Sehit Professor Doctor Ilhan Varank Education and Research Hospital, Istanbul, Turkey; 4 Nurten Bakan, Department of Anesthesiology and Reanimation, Health Sciences University, Sancaktepe Sehit Professor Doctor Ilhan Varank Education and Research Hospital, Istanbul, Turkey; 5 Nurettin Yiyit, Department of Thoracic Surgery, Health Sciences University, Sancaktepe Sehit Professor Doctor Ilhan Varank Education and Research Hospital, Istanbul, Turkey

**Keywords:** COVID-19, low molecular weight heparin, thrombosis

## Abstract

**Background and Objectives:**

COVID-19 might cause thrombosis in the arterial and venous system either directly or via indirect means such as cytokine storm or hypoxia. Enoxaparin might contribute to clinical recovery in COVID-19 patients, both by reducing the risk of thrombosis with anticoagulant effect and avoiding the cytokine storm with its anti-inflammatory effect. In this study, the clinical results of prophylactic enoxaparin usage in COVID-19 patients in our hospital were investigated.

**Methods:**

We retrospectively analyzed the patients who had hospitalized in our hospital with the diagnosis of COVID-19 between March 12 and April 17, 2020. Patients were divided into two groups according to their clinical status. Patients who were discharged to their home were in Group-I and were transferred to intensive care unit (ICU) were in Group-II. Patients’ demographics and laboratory examinations were compared between the groups. Then the effect of LMWH treatment in the rate of ICU transfer was evaluated.

**Results:**

There were 1216 hospitalized patients with COVID-19 in the study period. Increased age, levels of D-Dimer and fibrinogen and decreased hemoglobin, platelet, lymphocyte values were found to be statistically significantly risk factor for the need of ICU. Transfer rates of ICU were two times more in the patients who did not used enoxaparin and readmission after the discharge was higher in the patients who did not received enoxaparin in the hospital.

**Conclusion:**

Enoxaparin treatment in COVID-19 might be effective not only anticoagulant effect but also anti-inflammatory effect that decreased the risk cytokine storm. In the patients with COVID-19 disease, starting enoxaparin treatment in the earlier stage will decrease the risk of microthrombosis in vital organs and might improve the clinical outcomes.

## INTRODUCTION

Acute respiratory failure caused by COVID-19 was first reported in Wuhan, China in December 2019 and spread rapidly, causing the world health organization to be declared a pandemic in March 2020. The disease has caused more than three million people to be infected and more than 200,000 deaths in the world until May, 2020. The first case reported in Turkey on March 11. From this date to April 30, more than 120,000 people have infected and of 3,174 have been passed away.

It was reported that the only pathology in the respiratory system is not acute respiratory distress syndrome (ARDS) in COVID-19. In addition, microvascular thrombosis might be responsible for the pathogenicity of the virus.^1^,^2^ COVID-19 might cause thrombosis in the arterial and venous system by endothelial damage in the vascular bed directly or indirectly caused by infection and hypoxia.^1^,^3^ Inflammatory cytokines secreted during the disease might cause thrombosis by activating the coagulation cascade in different ways.^4^ Depending on the affected vascular bed, many systems might be damaged.

Severity of COVID-19 disease is associated with lymphopenia, prolonged coagulation tests, and increased D-Dimer.^5^ Abnormal blood clotting and thrombosis is a poor prognosis indicator in COVID-19.^4^ Coagulation tests were found to be increased and disseminated intravascular coagulation (DIC) was detected in most of the patients who died due to COVID-19.^6^ Thrombosis prophylaxis might help to prevent progression of the disease. In patients with mild symptoms, the risk of bleeding caused by thromboprophylaxis in the early stage of the disease is better than the time consuming until the evaluation of progressed disease in the overloaded health system.^2^ Therefore, thromboprophylaxis is recommended for all hospitalized patients with the suspicion of COVID-19.^2^,^7^

Enoxaparin, mostly preferred for thromboprophylaxis, reduces the release of T cell mediated cytokines as well as its known anti-inflammatory effect. It was declared to inhibit the release of IL-6 and IL-8 in vitro conditions.^8^ Respiratory decompensation caused by micro-thrombosis and abnormal immune responses might develop via activation of IL1B and IL6 in COVID-19.^9^ In this clinical status, enoxaparin might improve clinical condition both with its anticoagulant effect and preventing possible cytokine storm effect. In this study, the effects of enoxaparin treatment on clinical outcomes in the hospitalized patients with the diagnosis of COVID-19 were investigated.

## METHODS

We retrospectively analyzed the patients who had hospitalized with the diagnosis of COVID-19 infection in our hospital from March 12 to April 17, 2020. We included all patients who had hospitalized with the diagnosis of COVID-19 infection, in our hospital. Patients who did not accept to be hospitalized, hospitalized in the intensive care unit (ICU) at the admission and younger than 18 years old were excluded. Patients who were transferred to the ICU at the admission were excluded from the study, since we investigated the effects of thromboprophlylaxis in the clinical progression of the COVID-19. The study was approved by the Institutional Review Board and Turkish Ministry of Health. The data and laboratory examinations of the patients were gained from the medical records. Sepsis-induced coagulopathy (SIC) and DIC were calculated with the appropriate formula.^10^

The patients were divided into two groups according to their clinical course. Those who were discharged from the inpatient wards to their home constituted Group-I and those who were transferred to intensive care Group-II. Demographic information and laboratory findings between the groups were compared.

All patients with symptoms of infection and pulmonary involvement in computerized tomography (CT) were hospitalized inpatient wards for the treatment. PCR samples were taken at the admission. Patients were diagnosed of COVID-19 with clinical findings, PCR test and CT examination.

**Fig.1:**
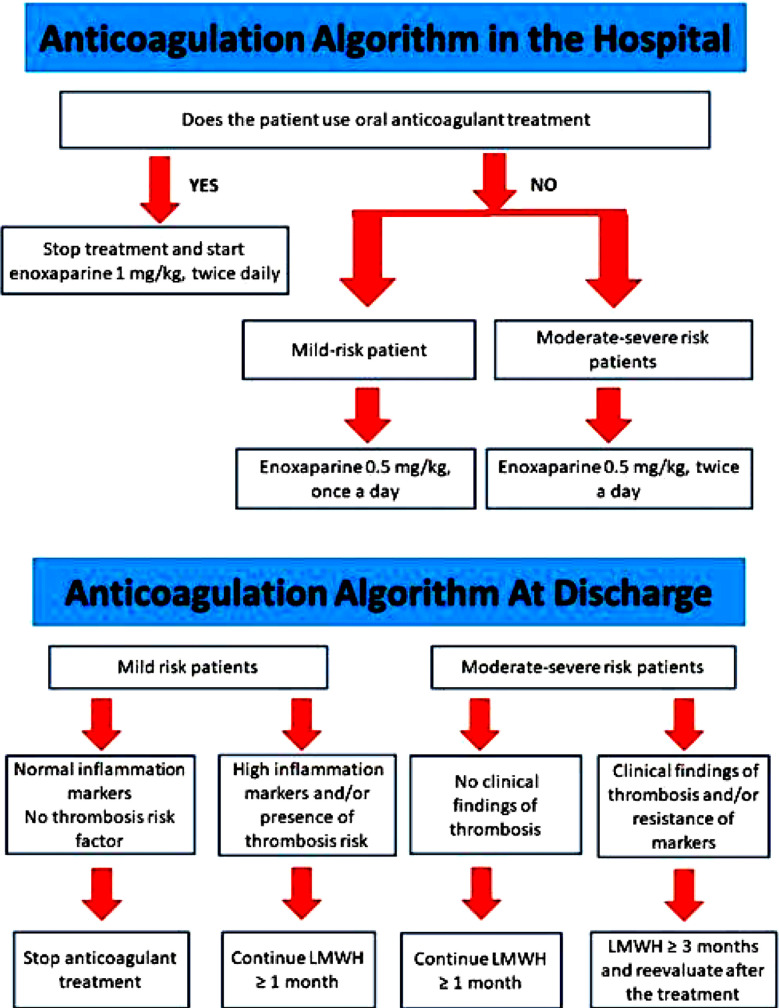
Treatment algorithm of anticoagulation.

Hydroxychloroquine was started routinely in the patients with COVID-19. Azithromycin and tamiflu were added to the treatment in patients with the suspicion of bacterial or influenza infection, respectively. Favipravir was started in patients whose respiratory distress continued and whose oxygen saturation fell below 90 during their follow-up. Patients whose oxygen saturation decreased below 90 with 2 lt / minute oxygen support and those whose respiratory symptoms progressed (tachypnea etc.) were transferred to the ICU. Patients with severe clinical condition, severe respiratory distress or needs to intubation were transferred to the ICU from the emergency service. Patients’ treatments were administered in accordance with the recommendations established by Turkey’s ministry of health sciences committee.^7^ In the earlier period, anticoagulant therapy was not routinely ordered. It was applied only in intensive care unit patients and immobile or risky patients (atrial fibrillation, history of thrombosis of pulmonary embolism) in March. With the guidance of updated recommendations of Turkey’s ministry of health, we had started to perform thromboprophylaxis in all hospitalized patients from the beginning of April.^7^ We designed and published an algorithm for thromboprophylaxis in our hospital (Fig.1). According to the algorithm, prophylactic dosage was increased to treatment dosage in the patients with severe clinical condition and with the suspicion of the thrombosis.

We investigated to protect health care professionals from the contamination. We investigated the effect of our thromboprophylaxis strategy, on the rate of ICU transfer and mortality, with comparing the patients who had received and who had not.

### Statistical Methods:

In the analysis of the data, SPSS (SPSS is free software; you can redistribute it and / or modify it under the terms of the GNU General Public License as published by the Free Software Foundation; either Version-3 of the License, or (at your option) any later version) and Microsoft Excel computer programs were used. Categorical variables are reported as frequency and percentage; continuous variables are reported appropriately as mean (SD). Chi-squared test, ANOVA and two sample t-test was performed for the evaluation. Logistic regression was used for risk analysis. The results were evaluated in the 95% confidence interval and p <0.05 significance level.

## RESULTS

A total of 1,251 patients were hospitalized with the diagnosis of COVID-19 infection at our hospital in the study period. There were 1,152 patients in Group-I and 99 patients in Group-II. Patients transferred to intensive care unit were statistically older and their hemoglobin, platelet, lymphocyte values were found to be statistically significantly decreased, and D-Dimer and fibrinogen values were increased (Table-I).

**Table-I:** Demographics and laboratory findings of patients.

	Group-I (n=1152)	Group-II (n=99)	*p*
Age (years)	50.12±15.9	59.63±17.3	<0.0001
Gender (M/F)	611/541	67/32	0.45
Hb (g/dL)	38.99±4.7	36.3±5.6	<0.0001
Htc (%)	13.16±1.7	12.25±2.2	<0.0001
Platelet (10^3^uL)	242.9±84.4	182.6±91.9	<0.0001
Lymphocyte (10^3^uL)	1.87±1.2	1.24±0.87	<0.0001
BUN (mg/dL)	14.99±9.8	30.34±23.7	<0.0001
Creatinin (mg/dL)	0.92±0.6	1.38±1.2	0.003
AST (u/L)	27.21±23.4	29.27±18.1	0.49
ALT (u/L)	28.42±25.8	36.59±28.5	0.03
INR	1.11±0.4	1.63±1.1	0.001
aPTT (sec)	28.98±4.5	29.25±5.8	0.78
D-Dimer (ug/mL)	1.23±3.4	3.38±3.1	<0.0001
Fibrinogen (mg/dL)	541±173.5	807.8±242.1	<0.0001
≥ 5 DIC score (n)	10 (1.4%)	19 (38.8%)	0.0001
≥ 4 SIC score (n)	14 (1.3%)	12 (19%)	0.0001

ALT=Alanine aminotransferase, aPTT= activated prothrombin time, AST=Aspartate aminotransferase, BUN=Blood urea nitrogen, DIC=Disseminated intravascular coagulation, F=Female, Hb= Hemoglobin, Htc= Hematocrit, INR=International normalized ratio, M=Male, SIC=Sepsis-induced coagulopathy.

Thromboprophylaxia was performed in 253 patients during the hospitalization period. Demographic and laboratory results were similar between the patients who were under the treatment of LMWH and were not. Eleven patients were re-hospitalized after discharge, and only two of them had used LMWH at their previous hospitalization. During the follow-up period of in-patient wards, seven patients transferred to the ICU urgently due to sudden respiratory distress. Only one of these patients was receiving LMWH treatment. There were 35 mortalities in the hospitalization period and only 10 of them under the treatment of LMWH. In the evaluation of the mortality in terms of LMWH treatment, no statistical difference was found (p = 0.19) (Table-II). In the analysis, it was seen that the need for ICU was two times more in patients who did not received LMWH. In the follow-up period, there was no deep venous thrombosis and bleeding complication in any of the patients.

**Table-II:** Comparison of demographics and laboratory findings of patients received LMWH.

	Patients Received LMWH (n=253)	Patients not Received LMWH (n=998)	*p*
Age (years)	51.2±16	50.48±16.2	0.53
Gender (M/F)	125 / 128	553 / 445	0.07
Platelet (10^3^uL)	238.4±92.2	240.08±84.2	0.79
Lymphocyte (10^3^uL)	1.81±0.93	1.84±1.2	0.67
D-Dimer (ug/mL)	1.51±2.95	1.37±3.69	0.67
Fibrinogen (mg/dL)	605±245.9	570.5±190.6	0.22
INR	1.16±0.4	1.14±0.5	0.67
aPTT (sec)	29.78±4.7	28.74±4.6	0.04
≥ 5 DIC score (n)	11 (5.2%)	18 (3.2%)	0.36
≥ 4 SIC score (n)	7 (3%)	19 (2.1)	0.38
*Clinical progress*			
Discharge to home (n)	222	930	0.008
Transfer to ICU (n)	21	43	
Mortality	10	25	0.19

aPTT = activated prothrombin time, DIC = Disseminated intravascular coagulation, F = Female, LMWH = Low molecular weight heparine, M = Male, ICU = Intensive care unit, INR = International normalized ratio, SIC = Sepsis-induced coagulopathy.

## DISCUSSION

This study predicts that enoxaparin treatment decrease the risk of thrombosis and cytokine storm in the infection with COVID-19. We compared the clinical outcomes and need of transfer to the ICU of the patients who applied enoxaparin with who did not. According to our results, enoxaparin treatment of the hospitalized patients due to COVID-19 decreases the need of the ICU. It also decreases the risk of re-admission and acute respiratory distress.

It was reported that, in COVID-19, the only pathology in the respiratory system is not ARDS, but also endothelial damage and microvascular thrombosis are effective.^1^, ^11^ Similar to SARS CoV, COVID-19 might cause thrombosis in the pulmonary arteries with small or medium diameter. The exact reason of hemostatic changes in COVID-19 has not been clarified. It might be caused by the direct effect of the virus or systemic inflammatory response syndrome.^1^, ^3^ COVID-19 might damage on many organs, including the lung, heart, vascular system, kidney and intestine via Angiotensin converting enzyme-2 (ACE2).^1^, ^3^, ^5^, ^12^, ^13^ It might aggravate platelet adhesion and aggregation by activating the renin-angiotensin system (RAS) when bound to ACE-2 receptors.^5^ Vascular accumulation of C5b-9, which is an important feature of many micro thrombotic syndromes such as anti-phospholipid antibody syndrome, atypical hemolytic uremic syndrome, purpura fulminans has also been found in COVID-19.^14^ This strengthens the idea that microvascular damage occurring in COVID-19 might be related to cytokine storm. Hydroxychloroquine, which is mostly preferred in the treatment of COVID-19, has antithrombotic effects by acting against anti-phospholipid antibodies. In addition, early awareness of abnormal coagulation parameters or prophylactic thromboprophylaxis at the time of diagnosis is important to avoid multiple organ damage.^2^ In our country, starting the treatment of hydroxychloroquine in all patients, although the symptoms are mild and patient will not be hospitalized; and ordering the enoxaparin to all the patients we hospitalized might be effective factors of our results.

**Table-III:** Evaluation of the effects of LMWH on the need of ICU.

	B	S.E.	Wald	df	Sig.	Exp(B)	95% C.I. for EXP(B)	

							Lower	Upper
Patients w/o treatment of LMWH	0.716	0.277	6.702	1	0.01	2.046	1.19	3.518

LMWH=Low molecular weight heparine, ICU=Intensive care unit.

Cytokine storm might play an important role in initiating and promoting thrombosis in arteries and veins.^9^ High levels of cytokines were detected in the plasma of critically ill patients with COVID-19.^1^,^15^ COVID-19 might lead to microthrombi in small vessels in the lung, kidney and heart by activation of IL-1B and IL-6 and inhibition of anti-thrombin 3 and might cause respiratory decompensation by abnormal immune response. Therefore, preventing cytokine storm through IL1-B and IL-6 might reduce the risk of thrombosis and alleviate the progression of the disease.^9^ Enoxaparin, which is used for thromboprophylaxis, has anti-inflammatory effect, which inhibit cytokine storm that might develop via IL6, in addition to its anticoagulant effect.^8^ In the fight against to COVID-19 in Turkey, the decrease in the number of patients necessitating ICU in a short time and the decrease in the number of cases earlier in comparison to other European countries might be related with starting LMWH treatment in all hospitalized patients.

DIC might be developed by damaged monocytes and endothelial cells in cytokine storm of sepsis.^4^,^6^ Increased incidence of DIC has been observed in patients with COVID-19 infection, and 70% of lost patients have been reported to meet the DIC criteria.^3^,^6^,^11^ Thromboprophylaxis with standard dosage has been reported to prevent the development of DIC in the ICU.^16^ Tang et al.^17^ reported that anticoagulant treatment contributed positively to the prognosis in patients with high SIC scores and increased D-Dimer levels in the evaluation of 449 patients. Similarly to the literature, DIC and SIC scores were found to be significantly higher in our patients who were transferred to ICU.

Abnormal coagulation parameters are reported in COVID-19.^11^ In the progression of the pulmonary disease, hypercoagulability develops and D-Dimer increases. Protrombin time is prolonged, fibrinogen and platelets levels are decreased in the severe disease. Lymphopenia was detected in patients who were referred to ICU.^4^,^15^ Mild thrombocytopenia, increased levels of D-Dimer and fibrin-derivated products (FDP), longer PT and activated protrombin time are characterized by higher mechanical ventilation rates, need for ICU and mortality.^5^, ^6^, ^18^ In addition, it was reported that fibrinogen and antithrombin levels decreased in the late period of hospitalization in the patients necessitating ICU.^6^ In our study, it was found that the lymphocyte and platelet values were decreased and INR, D-Dimer and fibrinogen values were increased in patients referred to the ICU.

Markedly elevated D-Dimer and FDP were reported in lost patients who admitted to hospital in the late period of the disease. This suggests that common coagulation activation, irregular thrombin production, impaired natural anticoagulants and fibrinolysis might be effective in the pathogenesis.^18^ D-dimer levels, higher than 1 mg/L at the admission, were shown to be associated with a marked increase in mortality risk.^5^,^11^ Dipyridamole treatment, a phosphodiesterase inhibitor, was reported to improve clinical outcome by reducing the D-Dimer levels and increasing the platelet and lymphocyte levels to normal values.^5^ According to our results, enoxaparin might have similar results with its anti-inflammatory effects.

Hospitalized patients have the risk of thrombosis due to reduced mobility, response to serious inflammation, severity of illness and underlying risk factors.^3^ It has been reported that 40% of hospitalized cases with COVID-19 have a risk of venous thrombosis.^3^ In a study conducted in China, in which 81 patients in the ICU were evaluated, 25% of patients developed venous thrombosis.^4^ However, prophylaxis was not applied in these patients. Thrombosis complication was reported in 1/3 of the ICU patients (27% in the venous system and 3.7% in the arterial system) in the evaluation of 184 patients in the Netherlands. Segmental or subsegmental pulmonary embolism was detected 80% of venous thrombosis.^16^ Therefore, they recommended high doses of thromboprophylaxis to all patients admitted to ICU. While Tang et al^17^ recommended thromboprophylaxis only in patients with high SIC score or D-Dimer level; Oudderk et al.^1^ recommended it all hospitalized patients with the diagnosis of COVID-19.

In COVID 19 patients, advanced examination such as CT angiography might be restricted due to both the risk of contamination and the clinical instability of the patient.^2^ The fact that the disease itself causes increased D-Dimer even in patients with stable course might delay the diagnosis of thrombosis.^2^ Diagnosis might be supported with lower extremity doppler ultrasonography or echocardiography at the bedside. However, overloaded patient circulation of pandemic period might delay this examinations.^2^ Early anticoagulation might prevent clot formation and reduce micro thrombus in COVID-19, thereby reducing the risk of major organ damage.^2^,^13^ In case of doubt of thrombosis, therapeutic dose might be switched to patients who cannot be examined.^1^ In thromboprophylaxis low molecular weight heparin is preferred to avoid repetitive aPTT measurement of standard heparin (UFH) to protect health professionals and decrease the use of protective equipment. We perform LMWH treatment with enoxaparin, in all patients who are hospitalized; and increase to the therapeutic dosage in patients with risky for thrombosis or with moderate / severe clinical condition

### Limitation of the Study:

The limitations of our study are the missing data which is nature of retrospective studies. Due to intense patient circulation during the pandemic period, patients were follow-upped by different branch physicians in different departments. Perspective of the different branches is one of the reason of the missing data and lack of standardization of the laboratory examinations. Number of examinations, requested in mild patients, might be limited to avoid kit restriction in the future, due to afraid of sudden load on the health system.

In COVID-19, micro-thrombosis that occurs directly or by endothelial damage induced by cytokine storm increase the severity of the disease. Application of thromboprophylaxis has started to increase in order to prevent thrombosis and its related complications with the increasing awareness of the disease. Enoxaparin might contribute to the mild course of the disease by reducing the progression of COVID-19, with anti-inflammatory effect in addition to the anicoagulant effect. Enoxaparin treatment period might be considered in the earlier stages of COVID-19 to decrease the risk of the progression.

### Author’s Contribution:

**MU** conceived, designed and did statistical analysis & manuscript writing.

**EA, ER** did data collection and editing of manuscript.

**NB, NY** revised article critically for important intellectual content.

## References

[1] Oudkerk M, Buller HR, Kuijpers D, van Es N, Oudkerk SF, McLoud TC (2020). Diagnosis, prevention, and treatment of thromboembolic complications in COVID-19:Report of the National Institute for Public Health of the Netherlands. Radiology.

[2] Obi AT, Barnes GD, Wakefield TW, Brown S, Eliason JL, Arndt E, Henke PE (2020). Practical diagnosis and treatment of suspected venous thromboembolism during COVID-19 pandemic. J Vasc Surg Venous Lymphat Disord.

[3] Bikdeli B, Madhavan MV, Jimenez D, Chuich T, Dreyfus I, Driggin E (2020). COVID-19 and thrombotic or thromboembolic disease:Implications for prevention, antithrombotic therapy, and follow-up. J Am Coll Cardiol.

[4] Cui S, Chen S, Li X, Liu S, Wang F (2020). Prevalence of venous thromboembolism in patients with severe novel coronavirus pneumonia. J Thromb Haemost.

[5] Liu X, Li Z, Liu S, Sun J, Chen Z, Jiang M (2020). Potential therapeutic effects of dipyridamole in the severely ill patients with COVID-19. 2020. Acta Pharm Sin B.

[6] Tang N, Li D, Wang X, Sun Z (2020). Abnormal coagulation parameters are associated with poor prognosis in patients with novel coronavirus pneumonia. J Thromb Haemost.

[7] COVID-19 (SARS-CoV-2 infection) guide (2020). Science Committee Work.

[8] Shastri MD, Stewart N, Horne J, Peterson GM, Gueven N, Sohal SS, Patel RP (2015). In-vitro suppression of IL-6 and IL-8 release from human pulmonary epithelial cells by non-anticoagulant fraction of enoxaparin. PLoS One.

[9] Barnes BJ, Adrover JM, Baxter-Stoltzfus A, Borczuk A, Cools-Lartigue J, Crawford JM (2020). Targeting potential drivers of COVID-19:Neutrophil extracellular traps. J Exp Med.

[10] Iba T, Levy JH, Warkentin TE, Thachil J, van der Poll T, Levi M, Scientific and Standardization Committee on DIC, and the Scientific and Standardization Committee on Perioperative and Critical Care of the International Society on Thrombosis and Haemostasis (2019). Diagnosis and management of sepsis-induced coagulopathy and disseminated intravascular coagulation. J Thromb Haemost.

[11] Zhu H, Rhee JW, Cheng P, Waliany S, Chang A, Witteles RM (2020). Cardiovascular complications in patients with COVID-19:Consequences of viral toxicities and host immune response. Curr Cardiol Rep.

[12] Zhang H, Penninger JM, Li Y, Zhong N, Slutsky AS (2020). Angiotensin-converting enzyme 2 (ACE2) as a SARS-CoV-2 receptor:Molecular mechanisms and potential therapeutic target. Intensive Care Med.

[13] Li T, Lu H, Zhang W (2020). Clinical observation and management of COVID-19 patients. Emerg Microbes Infect.

[14] Magro C, Mulvey JJ, Berlin D, Nuovo G, Salvatore S, Harp J (2020). Complement associated microvascular injury and thrombosis in the pathogenesis of severe COVID-19 infection:A report of five cases. Transl Res.

[15] Huang C, Wang Y, Li X, Ren L, Zhao J, Hu Y (2020). Clinical features of patients infected with 2019 novel coronavirus in Wuhan, China. Lancet.

[16] Klok FA, Kruip MJHA, van der Meer NJM, Arbous MS, Gommers DAMPJ, Kant KM (2020). Incidence of thrombotic complications in critically ill ICU patients with COVID-19. Thromb Res.

[17] Tang N, Bai H, Chen X, Gong J, Li D, Sun Z (2020). Anticoagulant treatment is associated with decreased mortality in severe coronavirus disease 2019 patients with coagulopathy. J Thromb Haemost.

[18] Frater JL, Zini G, d'Onofrio G, Rogers HJ (2020). COVID-19 and the clinical hematology laboratory. Int J Lab Hematol.

